# Niche partitioning by sympatric civets in the Himalayan foothills of Pakistan

**DOI:** 10.7717/peerj.14741

**Published:** 2023-02-21

**Authors:** Faraz Akrim, Tariq Mahmood, Jerrold L. Belant, Muhammad Sajid Nadeem, Siddiqa Qasim, Tashi Dhendup, Hira Fatima, Syed Afaq Bukhari, Asad Aslam, Humera Younis, Aamish Rafique, Zahid Ahmed Subhani, Shafqaat Ahmed Hashmi, Nadeem Munawar

**Affiliations:** 1 Department of Zoology, University of Kotli, Kotli, Azad Jammu and Kashmir, Pakistan; 2 Department of Wildlife Management, Pir Mehr Ali Shah Arid Agriculture University, Rawalpindi, Pakistan; 3 Department of Zoology Wildlife and Fisheries, Pir Mehr Ali Shah Arid Agriculture University, Rawalpindi, Pakistan; 4 Department of Fisheries and Wildlife, Michigan State University, East Lansing, Michigan, USA; 5 Wildlife Biology Program, Department of Ecosystem and Conservation Sciences, College of Forestry and Conservation, University of Montana, Missoula, Montana, USA; 6 Department of Wildlife & Ecology, University of Okara, Okara, Punjab, Pakistan

**Keywords:** Diet composition, Sympatric, Civet, Dietary breadth, Niche overlap

## Abstract

Niche overlap between sympatric species can indicate the extent of interspecific competition. Sympatric competing species can exhibit spatial, temporal, and dietary adjustments to reduce competition. We investigated spatial, temporal, and dietary niche overlap of sympatric Asian palm civet (*Paradoxurus hermaphroditus*) and small Indian civet **(***Viverricula indica*), in and around Pir Lasura National Park, Pakistan. We used remote cameras to determine the frequency and timing of detections to estimate spatial and temporal overlap, and prey remains from scats to estimate dietary overlap. We collected scat samples of Asian palm civet (*n* = 108) and small Indian civet (*n* = 44) for dietary analysis. We found low spatial (*O_ij_* = 0.32) and temporal (Δ = 0.39) overlap, but high dietary niche overlap (0.9) between these two civet species. Both civet species were detected at only 11 camera sites and small Indian civets were detected most frequently during 2:00–5:00 h and 8:00–10:00 h, whereas Asian palm civets detections were greatest during 20:00–2:00 h. The overall niche breadth of Asian palm civet was slightly narrower (L = 9.69, Lst = 0.31) than that of the small Indian civet (L = 10, Lst = 0.52). We identified 27 dietary items (15 plant, 12 animal) from scats of Asian palm civet including Himalayan pear (*Pyrus pashia;* 27%), Indian gerbil (*Tatera indica;* 10%), Rhesus monkey (*Macaca mulatta*; 4%), and insects (5%). Scat analysis of small Indian civets revealed 17 prey items (eight plant, nine animal) including Himalayan pear (24%), domestic poultry (15%), Indian gerbil (11%), and house mouse (*Mus musculus;* 5%). Both civet species consumed fruits of cultivated orchard species. Spatial and temporal partitioning of landscapes containing diverse foods appears to facilitate coexistence between Asian palm civets and small Indian civets.

## Introduction

The ecological niche refers to the “space” a particular species occupies in an ecosystem, including the physical environment, resources used, interactions with heterospecifics, and adaptations evolved for niche exploitation. A major factor that determines the niche of a species is competition (Caro & Stoner, 2003; Tilman, 2004). Species that compete for resources such as food can adjust their activity to avoid interactions by consuming different food items, in turn avoiding resource depletion shared between species (exploitative competition) or by aggression or territorial exclusion (interference competition) (Maurer, 1984; Amarasekare, 2002).

The niche of species can be broadly characterized in three dimensions including time, space, and diet or feeding, and ecologically similar, sympatric species have evolved different strategies to use these dimensions (Schoener, 1974). Sympatric species with similar niches adjust their use of resources to minimize niche overlap (Schoener, 1986), a phenomenon termed resource partitioning (Walter, 1991) or niche segregation. The segregation of niches can be an outcome of past competition among species (Lotka, 1978; Connell, 1980; Wootton, 1994; Krebs, 1999; Pianka, 2011) and facilitates species coexistence (Pianka, 1973); studies exploring different dimensions of niches are important to understand underlying mechanisms of co-existence (Jones & Barmuta, 2000).

Niche overlap between carnivore species can help to understand the extent of intraspecific competition and the intensity of interference competition (Allen et al., 2016). Competition is further reduced when species are distributed in different habitats or use the same habitat at different times of day (Schaller, 1972). Predators having similar diets often exhibit interspecific aggression to reduce competition, as they may encounter each other while searching for similar prey (Donadio & Buskirk, 2006). Many factors contribute to the coexistence of sympatric species including variations in the size of predators and hunting strategies used to capture prey species (Rosenzweig, 1966). The investigation of carnivore diets also can help to estimate dietary niche overlap between sympatric carnivore species (Akrim et al., 2019b; Hira et al., 2020) to characterize niche segregation.

The Asian palm civet (*Paradoxurus hermaphroditus*) is widely distributed in Asia from Afghanistan in the west to eastern China, including south-east Asian islands (Duckworth et al., 2016; Akrim et al., 2018, 2021). The small Indian civet (*Viverricula indica*) occurs in, Pakistan, Kashmir, Bangladesh, India, Nepal, Sri Lanka, China, mainland Southeast Asia, and in portions of Indonesia (Choudhury et al., 2015). These two species are sympatric in portions of their distribution ranges, but little is known of their ecology, particularly mechanisms that facilitate their coexistence in areas of sympatry.

The dietary niche overlap of many species of carnivores has been reported from Pakistan (Akrim et al., 2019a, 2019b; Hira et al., 2020); however, quantifying niche overlap in space, time, and diet has not been reported for any species in this country. We investigated spatial, temporal, and dietary niche overlap between sympatric Asian palm civet and small Indian civet to gain an understanding of mechanisms allowing coexistence.

### Study area

**Figure 1:**
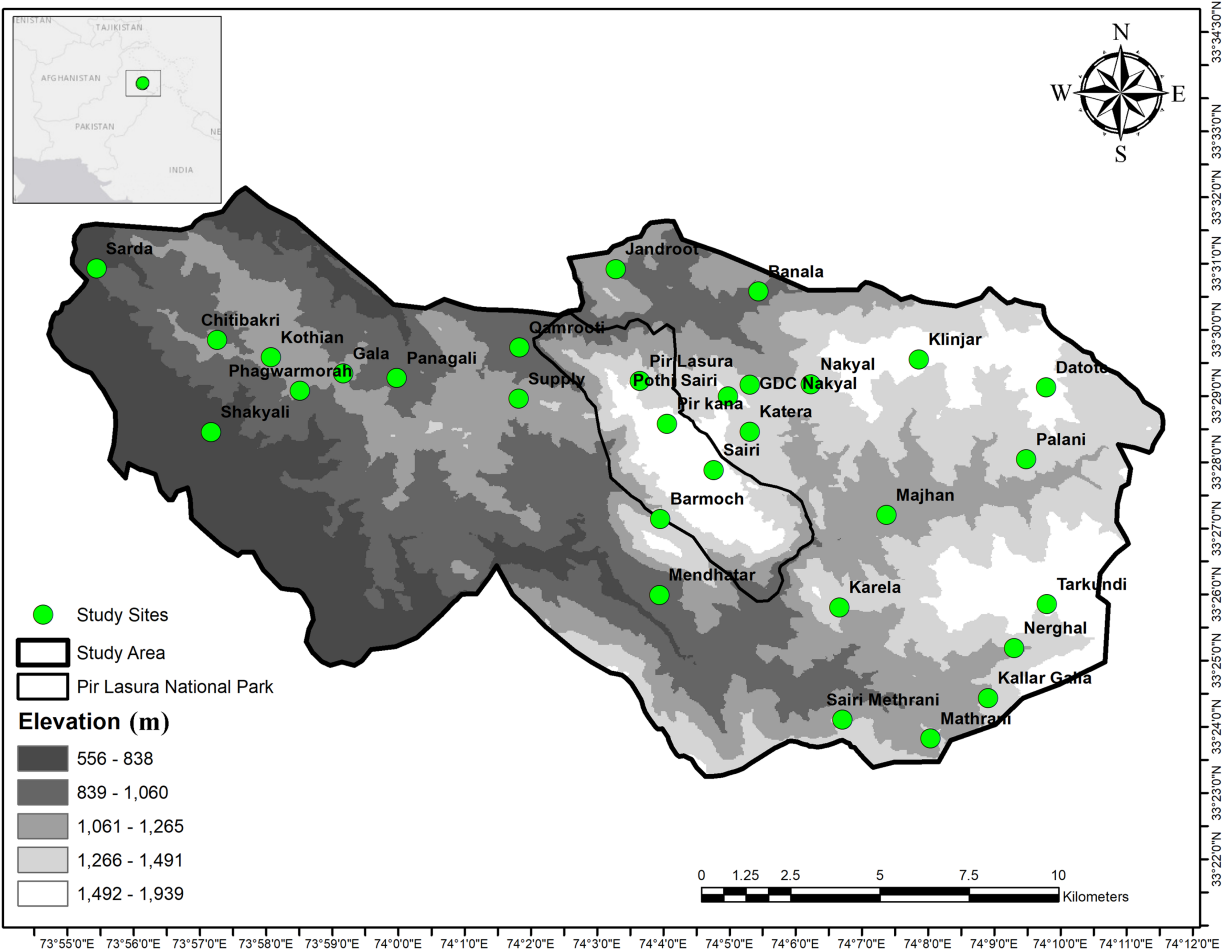
Map showing field signs of sympatric Asian Palm Civet (APC; *Paradoxurus hermaphroditus*), and small Indian Civet (*SIC*; *Viverricula indica*) in and around Pir Lasura National Park, Pakistan. Study sites (green circles) indicate locations of scats and cameras.

We conducted this study in and around Pir Lasura National Park (PLNP; 33°25.92–33° 29.31 N; 74°05.64 74°03.02 E), District Kotli, Azad Jammu and Kashmir, Pakistan (Fig. 1). The park comprises 1,580 ha and our overall study area was 17,183 ha. Elevations range from 1,000 to 2,000 m above sea level with total annual precipitation of 1,250 mm (Amjad et al., 2017). Lower elevations include subtropical pine (*Pinus* spp.) forests, with higher elevations having sub-tropical dry evergreen forests. Local communities grow corn and millet during summer, wheat during winter (Akrim et al., 2021), and vegetables for household use. Mammal species in the study area include the common leopard (*Panthera pardus*), Asiatic jackal (*Canis aureus*), red fox (*Vulpes vulpes*) (Akrim et al., 2019a), small Indian mongoose (*Herpestes auropunctatus*), Indian grey mongoose (*Herpestes edwardsii*) (Akrim et al., 2019b), Rhesus monkey (*Macaca mulatta*), barking deer (*Muntiacus muntjak*), Indian pangolin (*Manis crassicaudata*) and Kaleej pheasant (*Lophura leucomelanos*) (Akrim et al., 2017, 2018).

## Methods

### Sampling

We conducted fieldwork from 2016 to 2021. Written permission was obtained from the ethical committee of the Department of Wildlife Management PMAS AAUR (PMAS-AAUR/2646) to conduct the field surveys. We collected civet scats during 2016–2017, traversing 1–4 km of roads and trails in the study area each month. We identified scats of species based on morphological characteristics involving shape, color, odor, dimensions (length and diameter), number of segments, and physical appearance (Seton, 1925; Jackson & Hunter, 1995; Wemmer et al., 1996) as well as other evidence (*e.g*., tracks) followed by genetics. We stored samples separately in bags labeled with the location and date of collection.

We deployed 30 cameras during 2018–2021 and selected camera sites based on the presence of civet signs while also considering the potential for camera theft (Mugerwa et al., 2017). We removed vegetation in front of the camera to reduce false triggers. At each site, we installed a camera (Bushnell HD Trophy Camera, Model 11953; Bushnell, Overland Park, KS, USA or UOVision UV557, Shenzen, China) on a tree 60 cm above the ground (Tanwar, Sadhu & Jhala, 2021). Cameras were operational at each site for 15 days.

### Spatial niche overlap

We recorded the location, date, and time of detection from camera images for both civet species. To keep our detections independent, camera trap data of multiple captures of species within 30 min intervals were excluded from the analysis. To estimate spatial overlap, we summed the frequency of detections for each species at each site and used Pianka’s niche overlap index (Pianka, 1973). The values of Pianka’s index range from 0 indicating no overlap to 1 indicating overlap (Pianka, 1973), derived using the equation:

${o_j}k = \displaystyle{{\mathop \sum \limits_i^n {p_{ij}}{p_{ik}}} \over {\sqrt {\mathop \sum \limits_i^n p_{ij}^2\mathop \sum \limits_i^n p_{ik}^2} }}$

where *p*_*ij*_ and *p*_*ik*_ represents the relative frequency of photo capture by camera traps at site *i* for species *j* or *k*.

### Temporal niche overlap

We estimated the temporal overlap between the Asian palm civet and the small Indian civet using the package Overlap (Ridout & Linkie, 2009) in program R (R Core Team, 2021). We converted the time of each capture into radians to obtain circular distributions of temporal data (Meredith & Ridout, 2014; Rowcliffe et al., 2014) and used the coefficient of overlap (Δ) to quantify temporal activity overlap between civet species (Ridout & Linkie, 2009). The coefficient of overlap measures the degree of overlap using circular kernel density estimates by incorporating the minimum density function from the two samples of detection data compared at each point in 24-h time, with the area falling under the density curves indicating overlap (Ridout & Linkie, 2009). The value of the coefficient of overlap indicated the intensity of temporal niche overlap 0 indicating no overlap and 1 indicating complete overlap (Ridout & Linkie, 2009; Linkie & Ridout, 2011). Since number of observations of both species were >75 we used dhat4 (Δ_4_) estimator to compute niche overlap. We categorized temporal niche overlap based of value of coefficient of overlap as low (≤50), moderate (50 < ∆ ≤ 75) and high (∆ > 75) (Monterroso, Alves & Ferreras, 2014). Based on sunrise and sunset time day was divided into three periods 6:00–18:30 h as day, 20:30 to 4:00 h as night, 4:00–6:00 h as dawn and 18:30–20:30 h as dusk.

### Dietary niche overlap

The fecal DNA was extracted using QIAamp DNA Stool Mini Kits (Qiagen, INC., Valencia, CA, USA) using 12S/V5 primer (Riaz et al., 2011). We used negative control to keep track of cross-contamination during extraction (Beja-Pereira et al., 2009). The PCR for all scat samples was carried out in a total volume of 50 µL. The 3130 XL genetic analyzer was used to read DNA sequences and all sequences were blasted on NCBI blast for species identification. To estimate diet, we oven-dried the scats before rinsing them in warm water to remove dust and mucus, then allowed the remains to air dry. We manually separated scat contents by food types (Mahmood, Niazi & Nadeem, 2013; Mukherjee, Goyal & Chellam, 1994). We first washed hair in carbon tetrachloride for 15–20 min, then prepared whole-mount slides using transparent nail polish (Lavoie, 1971). We used cuticular scale patterns of hair under light microscopy and reference materials to identify mammalian prey remains to species. We also used reference collection material to identify other remains found in scats (*e.g*., bones, feathers, invertebrate exoskeletons, and seeds). We tabulated the data as relative frequency of occurrence (*i.e*., number of occurrences of a food item in all scats/total number of occurrences of all food items in all scats × 100) for each food type. We did not estimate biomass consumed because of biases among food types (Chakrabarti et al., 2016; Lumetsberger et al., 2017).

We measured the dietary niche breadth for each civet species using niche breadth (L) and standardized Levins index (0−1) (*Lst*) (Levins, 1968; Colwell & Futuyma, 1971) as follows:

$L = {\left( {\mathop \sum \limits_{i = 1}^n \rho _{\dot l}^2} \right)^{ - 1}} \rm and$

$Lst = \displaystyle{{L - 1} \over {n - 1}}$

where, *p*_*i*_ represents the relative frequency of each dietary item, *i* and *n* are the number of dietary items. The *Lst* is standardized niche breadth, and the value of *Lst* ranges between 0 (narrow niche breadth) to 1 (broader niche breadth).

Using the frequency of occurrence of each food item in the diet of both civet species we computed dietary niche overlap using Pianka’s index, with values that range from 0 indicating no dietary overlap between two civet species to 1 representing complete dietary overlap in EcoSimR package in R software (Pianka, 1973; Gotelli et al., 2015). We also calculated the prey species diversity index (H′), prey richness (S), and prey evenness (E) for each civet species. We calculated prey diversity (H′) by the following formula:

${\bf H^\prime}=-\sum{[pi \times In\;pi],}$

where pi is the prey index. We calculated prey species evenness (E) as:

${\bf E}={\rm H^\prime/In\;of\;S},$

where S is the number of prey species (richness) and H′ is the diversity index.

## Results

We detected Asian palm civets at 7 of 30 sites (98 detections), small Indian civets at 11 of 30 sites (1,321 detections), and neither species at 19 sites. Spatial niche overlap between the two civet species was low (*O*_*ij*_ = 0.32; Table S1).

**Figure 2:**
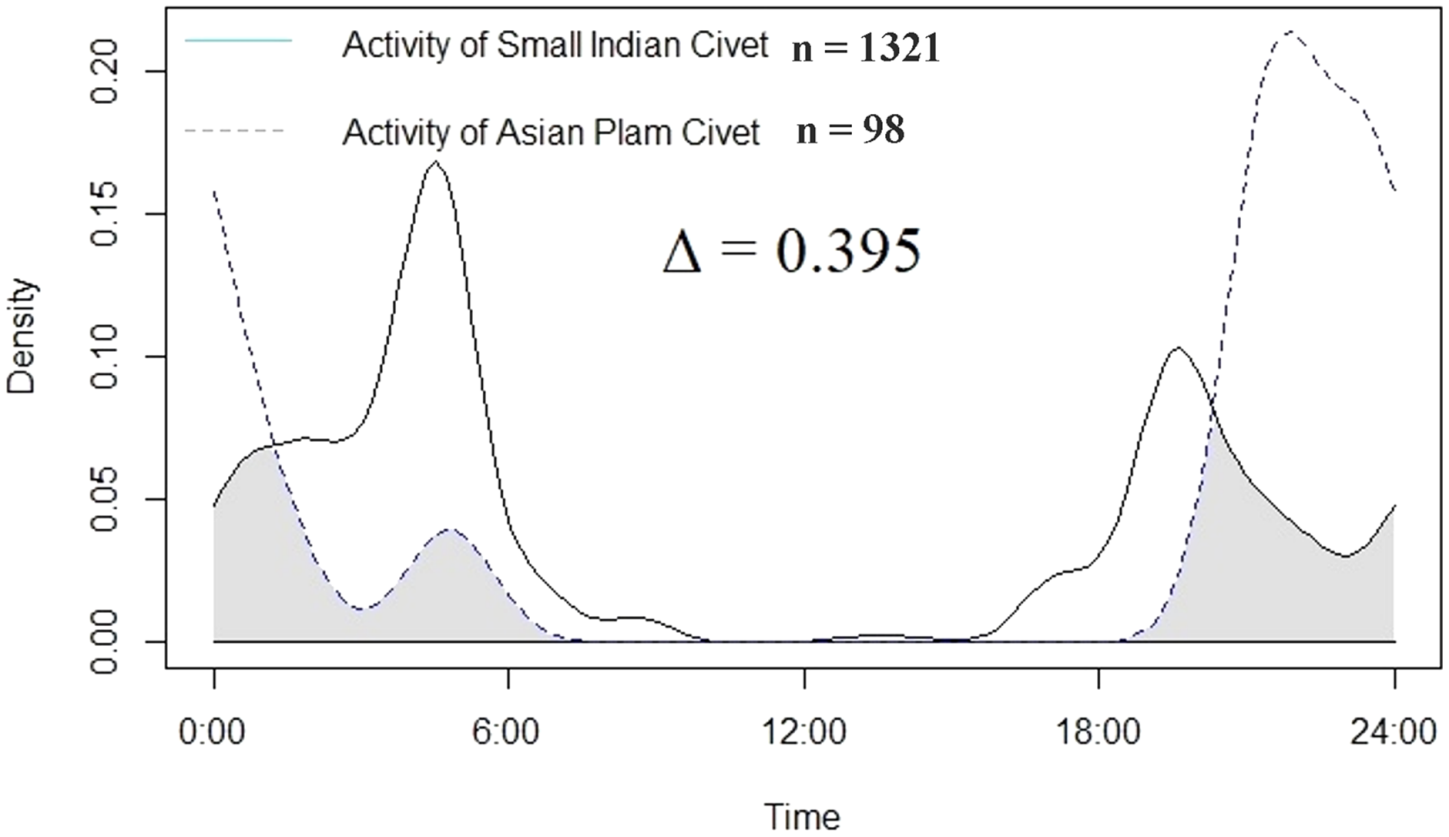
Temporal activity overlap of two civet species in and near Pir Lasura National Park, Pakistan.

Temporal overlap between the Asian palm civet and small Indian civet was low (Δ_4_ = 0.39; Fig. 2). The activity of small Indian civet was greatest during 2:00–5:00 h, followed by 20:00–22:00 h, whereas the activity of Asian palm civet was greatest during 19:00–1:00 h.

We confirmed the identity of 108 scats of Asian palm civet and 44 scats of small Indian civet species. We observed high dietary niche overlap (0.9) between Asian palm civet and small Indian civet. Food items recovered from each scat of Asian palm civet ranged from one to five and for small Indian civet ranged from one to three. Asian palm civets consumed 27 different food items, including 15 plants (53.4% of diet) and 12 animals (44.6%). Wild animal prey was more frequently consumed (33.7%, nine species) than domestic prey (10.9%, three species). Wild mammals consumed included Indian gerbil (9.8%), Rhesus monkey (4.2%), and Norway rat (3.6%). The frequency of invertebrate consumption was low with insects and snails contributing 4.7% and 3.6% of the diet, respectively. Among domestic species, poultry was most common (6.7%) followed by cattle (3.6%). Among plant species consumed, the wild Himalayan pear was most common (27.5%), followed by blackberry (5.7%), and jujube (5.2%, Table 1).

**Table 1:** Frequency of occurrence (FO) and relative frequency of occurrence (RFO) of food items in scats of Asian palm civet (*n* = 108) and small Indian civet (*n* = 44) in and near Pir Lasura National Park, Pakistan, 2014–2017.

	Asian palm civet		Small Indian civet	
**Food item**	**FO**	**RFO**	**FO**	**RFO**
**Wild animal**				
Indian Gerbil (*Tatera indica*)	19	9.8	7	11.3
Rhesus Monkey (*Macaca mulatta*)	8	4.2	0	0.0
Norway rat (*Rattus norvegicus*)	7	3.6	2	3.2
House mouse (*Mus musculus*)	6	3.1	3	4.8
Roof or house rat (*Rattus rattus*)	4	2.1	1	1.6
Desert hare (*Lepus nigricollis dayanus*)	5	2.6	0	0.0
Himalayan bulbul (P*ycnonotus leucogenys*)	0	0.0	2	3.2
House sparrow (*Passer domesticus*)	0	0.0	1	1.6
Amphibians	0	0.0	2	3.2
Snail (Cornu spp.)	7	3.6	0	0.0
Insects (Orthoptera) Grasshopper	7	3.6	1	1.6
Insects (Coleoptera) beetle	2	1.0	0	0.0
Sub-total	65	33.7	19	30.7
Domestic animal				
Chicken (*Gallus gallus domesticus*)	13	6.7	9	14.5
Cattle (*Bos taurus*)	7	3.6	0	0.0
Sheep (*Ovis aries*)	1	0.5	0	0.0
Sub-total	8	10.9	9	14.5
Plants				
Wild Himalayan pear (Dhandali) (*Pyrus pashia*)	53	27.5	15	24.2
Blackberry (Aakharay) (*Rubus fruticosus*)	11	5.7	5	8.1
Jujube (*Ziziphus oxyphylla*)	10	5.2	2	3.2
Olea (Kov) (*Olea ferruginea*)	6	3.1	2	3.2
Jujube (aka bara bair) (*Ziziphus jujube*)	4	2.1	0	0.0
Date-plum (*Diospyros lotus*)	4	2.1	1	1.6
Loquat (*Eriobotrya japonica*)	0	0.0	3	4.8
Loonder grass (*Themeda anathera*)	2	1.0	0	0.0
Apple (*Pyrus malus*)	2	1.0	3	4.8
Apricot (Khobani) (*Prunus amrmeniaca*)	2	1.0	0	0.0
Wild fig (*Ficus carica*)	2	1.0	0	0.0
Watermelon (*Citrullus lanatus*)	2	1.0	1	1.6
Coriander (Dhania) (*Coriandrum sativum*)	2	1.0	0	0.0
Indian ash tree (Kumlo) (*Lannea coromandelica*)	1	0.5	0	0.0
Orange (*Citrus reticulate*)	1	0.5	0	0.0
Wheat (*Triticum aestivum*)	1	0.5	0	0.0
Sub-total	103	53.4	32	51.6
Grit	3	1.6	1	1.6
Artificial matter	1	0.5	1	1.6

Small Indian civets consumed 17 different food items including plant matter (51.6%, eight species) and animal matter (45.2%, nine species) (Table 1). Wild prey consumption (30.7%; eight species) was greater than that of domestic prey (14.5%, one species). Mammals contributed (21.0%) to the wild diet followed by birds (4.8%) and amphibians (3.2%). Among wild prey, consumption of Indian gerbil was greatest (11.29%), followed by house mouse (4.84%). Among plants, the wild Himalayan pear was most frequently consumed (24.2%), followed by blackberry (8.1%), and apple and loquat (4.8% each).

The overall dietary niche breadth of Asian palm civet was slighter narrower (L = 9.69, Lst = 0.31) than that of the small Indian civet (L = 10, Lst = 0.52) and high dietary niche overlap occurred between two species (0.9). Prey species diversity (H′) and prey species richness of Asian palm civet (2.78 and 29, respectively) were greater than those of the small Indian civet (2.53 and 19), whereas prey species evenness was greater for small Indian civet (0.86) than for Asian palm civet (0.82).

## Discussion

We provide the first study demonstrating spatial, temporal, and dietary niche overlap between Asian palm civet and small Indian civet. We found limited spatial and temporal overlap but identified a high degree of omnivory and dietary overlap for these species, with includes the use of domestic animals and plants suggesting partitioning of resources other than food (space and time) facilitates their coexistence.

Our results indicated spatial segregation between species, also finding reduced use of sites by Asian palm civets where the frequency of detection of small Indian civets was high. Asian palm civet is semi-arboreal while small Indian civet is terrestrial resulting in vertical spatial segregation between them (Roberts, 1997; Su & Sale, 2007). All the camera traps were located on ground level, and this might be a logical reason for low detections of Asian palm civet. Both civet species occurred on the ground however Asian palm civet also utilized the upper strata of the forests (Su & Sale, 2007). The semi-arboreal adaptation of the Asian palm civet partitions its spatial niche vertically with the small Indian civet facilitating both species to coexist in the same landscape (Roberts, 1997). According to the theory of limiting similarity (MacArthur & Levins, 1967), sympatric competing species should segregate in at least one niche dimension (*i.e*., space, time, or diet in this study). Our study area contains patchy distributions of human populations with corresponding habitat fragmentation and could provide this civet species opportunity to alter space use to enable coexistence in the same landscape.

We recorded low temporal niche overlap between two civet species. Many studies have demonstrated the abilities of carnivores to adjust their biological rhythms with local conditions for temporal segregation facilitating coexistence (Di Bitetti et al., 2010). Research conducted in Borneo, Malaysia showed no temporal niche segregation between three civet species (Nakabayashi et al., 2021). Our findings suggest one or both species showed different temporal activity patterns. During our study, small Indian civets demonstrated peak activity immediately following a decline in Asian palm civet activity. Similar findings were reported from Myanmar that the temporal activity of Asian palm civet and small Indian civet were different from each other (Su & Sale, 2007). The small Indian civet exhibited nocturnal behavior though it was frequently detected during crepuscular periods. Activity of small Indian civet during midnight and soon after the sunset time was reported in Western Ghats, India (Sanghamithra & Nameer, 2021). In contrast, the Asian palm civet showed high nocturnal activity as compared to the small Indian civet.

The only previous study on civet diets from Pakistan noted that Asian palm civets live near villages and subsisted on rats and mice while also attracted to food orchards and plantations (Roberts, 1997). Roberts (1997) further noted that small Indian civets consumed small mammals, lizards, and birds. However, the diets of both species were determined from limited field observations and anecdotal reports from local people. The omnivorous Asian palm civet plays an important ecological role as predator, prey, and seed disperser (Nakashima & Sukor, 2010; Nakashima et al., 2010; Nakashima, Inoue & Inoue-Murayama, 2010; Aroon et al., 2012). In our study, consumption of plant matter was high in the diet of Asian palm civet, similar to previous studies (Rabinowitz, 1991; Corlett, 1998; Nakashima et al., 2010), but less than civets from Nepal and India (82–91%) (Krishnakumar & Balakrishnan, 2003, Jothish, 2011). Variation in percentage fruit consumption could be caused by several factors including spatio-temporal variation in fruit (and alternate prey) availability as well as differences in methodologies for diet estimation. In Myanmar, the frequency of fruits in the diet was greater during the rainy season than in winter (Rabinowitz, 1991; Kitamura et al., 2002) and Asian palm civets consumed more mammalian prey when fruit availability was low (Aroon et al., 2012).

The frequency of occurrence of plants we observed was also high in the diet of small Indian civet. Small Indian civets are omnivorous, and may rely on fruit when available, but also consume rodents, birds, invertebrates, and non-fruit plant matter (Wang & Fuller, 2003). Wang, Sheng & Lu (1976) reported high consumption of rodents (80%) and insects (23%) by small Indian civets. Similarly, small Indian civets in Taiwan consumed large amounts of rodents and shrews (40%), insects (95%), and earthworms (67%) (Chuang & Lee, 1997). In contrast, the total animal matter consumed by small Indian civets in this study comprised only 45% of their diet. Variation in the diet of small Indian civets suggests considerable dietary flexibility. Further, our study along with previous works (*e.g*., Corlett, 2011; Dehaudt et al., 2022) suggest that small Indian civets may play an important role in the seed dispersal of some wild plant species. However, the consumption of domestic fruits in orchards can result in conflicts with humans and the potential illegal killing of civets (F. Akrim, personal observation, 2017). The diversity and richness of prey species consumed by Asian palm civets were greater than that of small Indian civets. However, prey species evenness and niche breadth between species were similar. Overall, this indicates that both civet species exploited a wide range of prey species.

The overall niche breadth of the Asian palm civet was slighter narrower (Lst = 0.31) than that of the small Indian civet (Lst = 0.52). Previous studies reported a niche breadth of 0.412 for Asian palm civet (Aroon et al., 2012). Chuang & Lee (1997) reported that the dietary breadth of small Indian civet was 4.46 in northern Taiwan whereas in China their reported niche breadth was 2.58 comprised of nine species (Wang & Fuller, 2003). High niche overlap between Asian palm civets and small Indian civets in this study (0.9) indicates that both species consumed similar foods and might compete when resources are limited, resulting in interspecific completion.

We demonstrated high dietary niche overlap between Asian palm civets and small Indian civets that partitioned resources in space and time to facilitate coexistence. All our camera traps were placed on ground level for 15 days at each site and due to the semi-arboreal adaption of Asian palm civet, the detections were low as compared to small Indian civet which is a limitation of the current study. Further studies of the niche overlap among sympatric Asian palm civets and small Indian civets under varying conditions such as seasonal variation, responses to human disturbance and considering adaptations of both species (terrestrial and semi-arboreal) can further our understanding of underlying mechanisms facilitating coexistence.

## Supplemental Information

10.7717/peerj.14741/supp-1 Supplemental Information 1 Spatial overlap between Asian palm civet (APC) and small Indian civet (*SIC*) estimated by Pianka index in and near Pir Lasura National Park, Pakistan.

10.7717/peerj.14741/supp-2 Supplemental Information 2 Raw data of diet composition analysis.

## Additional Information and Declarations

### Data Availability

The following information was supplied regarding data availability: Raw data is available as a Supplemental File.
