# Supplementary material for: Niche partitioning by sympatric civets in the Himalayan foothills of Pakistan
Source: PeerJ. 2023 Feb 21;11:e14741. doi: 10.7717/peerj.14741 (PMC9951805; doi:10.7717/peerj.14741)
Supplement: Supplemental Information 1 [file peerj-11-14741-s001.docx]

Supplementary Table 1: Spatial overlap between Asian palm civet (APC) and small Indian civet (SIC) estimated by Pianka index in and near Pir Lasura National Park, Pakistan.

| **Study Sites** | **Photos of APC** | **Photo of**  **SIC** | **Proportion of APC** | **Proportion of SIC** |
| --- | --- | --- | --- | --- |
| Phagwarmorah | 3 | 6 | 0.03 | 0.0045 |
| Pir Kana | 24 | 3 | 0.24 | 0.0023 |
| Sarda | 0 | 12 | 0 | 0.009 |
| Panagali | 27 | 47 | 0.27 | 0.035 |
| Shakyali | 0 | 204 | 0 | 0.154 |
| Chitibakri | 0 | 152 | 0 | 0.115 |
| Karela | 0 | 2 | 0 | 0.0015 |
| Majhan | 17 | 34 | 0.17 | 0.0257 |
| Sairi | 13 | 39 | 0.13 | 0.0295 |
| Nakyal | 11 | 550 | 0.11 | 0.416 |
| Kothian | 3 | 272 | 0.03 | 0.206 |
| **Total** | **98** | **1321** | **1** | **1** |
|  |  |  | ***O_ij_* (overlap)** | **0.32** |
